# Supplementary material for: Meta-Analysis and Gene Set Enrichment Relative to ER Status Reveal Elevated Activity of MYC and E2F in the “Basal” Breast Cancer Subgroup
Source: PLoS One. 2009 Mar 9;4(3):e4710. doi: 10.1371/journal.pone.0004710 (PMC2650420; doi:10.1371/journal.pone.0004710)
Supplement: Dataset S1 — GSEA results from first screen. The file contains a zipped archive of the HTML results of the second GSEA screen. Reports for enriched or depleted datasets may be accessed from the “index.html” link within the my_analysis.GseaPreranked.1219975950625.rpt.zip ZIP archive. Enrichment plots are available for each of the 50 most enriched and depleted gene sets. (13.32 MB ZIP) [file pone.0004710.s001.zip › my_analysis.GseaPreranked.1219975950625.rpt.zip!//CHR14Q22.html]

Details for gene set CHR14Q22[GSEA]

|  || Dataset | zSco.ERneg.to.ERpos\_collapsed |
| Phenotype | NoPhenotypeAvailable |
| Upregulated in class | na\_neg |
| GeneSet | CHR14Q22 |
| Enrichment Score (ES) | -0.5031716 |
| Normalized Enrichment Score (NES) | -1.9791327 |
| Nominal p-value | 0.0 |
| FDR q-value | 0.009935138 |
| FWER p-Value | 0.226 |
Table: GSEA Results Summary

  

Fig 1: Enrichment plot: CHR14Q22      
 Profile of the Running ES Score & Positions of GeneSet Members on the Rank Ordered List

  

| PROBE | GENE SYMBOL | GENE\_TITLE | RANK IN GENE LIST | RANK METRIC SCORE | RUNNING ES | CORE ENRICHMENT || 1 | DLG7 |  |  | 521 | 4.306 | 0.0069 | No |
| 2 | WDHD1 |  |  | 1576 | 2.872 | -0.0416 | No |
| 3 | CDKN3 |  |  | 1827 | 2.674 | -0.0318 | No |
| 4 | SAMD4A |  |  | 2082 | 2.462 | -0.0245 | No |
| 5 | SNW1 |  |  | 2259 | 2.344 | -0.0126 | No |
| 6 | SYNE2 |  |  | 3041 | 1.912 | -0.0509 | No |
| 7 | PLEKHC1 |  |  | 3571 | 1.675 | -0.0728 | No |
| 8 | SNAPC1 |  |  | 4062 | 1.488 | -0.0937 | No |
| 9 | PGF |  |  | 4227 | 1.420 | -0.0908 | No |
| 10 | SIX6 |  |  | 4280 | 1.400 | -0.0797 | No |
| 11 | NAT12 |  |  | 4854 | 1.205 | -0.1100 | No |
| 12 | ZFP36L1 |  |  | 5368 | 1.023 | -0.1376 | No |
| 13 | GCH1 |  |  | 5810 | 0.875 | -0.1614 | No |
| 14 | PTGDR |  |  | 6405 | 0.682 | -0.1989 | No |
| 15 | ERO1L |  |  | 6926 | 0.510 | -0.2325 | No |
| 16 | C14ORF105 |  |  | 7359 | 0.364 | -0.2612 | No |
| 17 | C14ORF118 |  |  | 7959 | 0.120 | -0.3050 | No |
| 18 | C14ORF138 |  |  | 7970 | 0.117 | -0.3045 | No |
| 19 | PTGER2 |  |  | 8494 | -0.074 | -0.3431 | No |
| 20 | EXOC5 |  |  | 8580 | -0.116 | -0.3482 | No |
| 21 | C14ORF32 |  |  | 8887 | -0.251 | -0.3686 | No |
| 22 | FBXO34 |  |  | 9108 | -0.366 | -0.3812 | No |
| 23 | TXNDC1 |  |  | 9992 | -0.836 | -0.4387 | No |
| 24 | C14ORF166 |  |  | 10669 | -1.295 | -0.4758 | No |
| 25 | SDCCAG1 |  |  | 11034 | -1.598 | -0.4860 | Yes |
| 26 | C14ORF101 |  |  | 11121 | -1.681 | -0.4745 | Yes |
| 27 | L2HGDH |  |  | 11163 | -1.714 | -0.4592 | Yes |
| 28 | CNIH |  |  | 11576 | -2.108 | -0.4676 | Yes |
| 29 | DACT1 |  |  | 11756 | -2.272 | -0.4567 | Yes |
| 30 | PPM1A |  |  | 12195 | -2.837 | -0.4593 | Yes |
| 31 | GMFB |  |  | 12201 | -2.842 | -0.4292 | Yes |
| 32 | C14ORF108 |  |  | 12278 | -2.938 | -0.4034 | Yes |
| 33 | TIMM9 |  |  | 12307 | -2.970 | -0.3736 | Yes |
| 34 | KTN1 |  |  | 12476 | -3.222 | -0.3517 | Yes |
| 35 | BMP4 |  |  | 12783 | -3.824 | -0.3337 | Yes |
| 36 | ARID4A |  |  | 12806 | -3.906 | -0.2935 | Yes |
| 37 | DAAM1 |  |  | 12945 | -4.313 | -0.2576 | Yes |
| 38 | HSPA2 |  |  | 13023 | -4.529 | -0.2149 | Yes |
| 39 | ACTR10 |  |  | 13166 | -5.143 | -0.1704 | Yes |
| 40 | SYNJ2BP |  |  | 13207 | -5.444 | -0.1150 | Yes |
| 41 | KIAA0831 |  |  | 13219 | -5.578 | -0.0560 | Yes |
| 42 | CGRRF1 |  |  | 13248 | -5.923 | 0.0054 | Yes |
Table: GSEA details [plain text format]

  

Fig 2: CHR14Q22: Random ES distribution      
 Gene set null distribution of ES for **CHR14Q22**
